# Hypothermia is Associated With Poor Prognosis in Hospitalized Patients With Severe COVID-19 Symptoms

**DOI:** 10.7759/cureus.14526

**Published:** 2021-04-16

**Authors:** Yousef Maait, Marc El Khoury, Lee McKinley, Anthony El Khoury

**Affiliations:** 1 School of Clinical Medicine, University of Cambridge, Cambridge, GBR; 2 Intensive Care Unit, Indiana University School of Medicine, Evansville, USA; 3 Internal Medicine, Indiana University School of Medicine, Evansville, USA

**Keywords:** covid-19, hypothermia, intensive care unit

## Abstract

Rationale

Hypothermia forms a part of the diagnostic criteria for Systemic Inflammatory Response Syndrome (SIRS), National Early Warning Score (NEWS) and has repeatedly been shown to be associated with worse outcomes when compared to normothermic and hyperthermic patients with sepsis. We evaluate whether this is the case in COVID-19 patients.

Objective

To determine whether there is an association between hypothermia and worse prognosis in COVID-19 patients in the intensive care unit.

Methods

Retrospective study of a cohort of patients (n = 57) admitted to the intensive care unit of a community hospital with a positive test for COVID-19.

Measurements

Data relating to mortality, comorbidities and length of stay was recorded from electronic medical records for each patient. Hypothermia was defined as ≥2 recorded body temperatures of less than 96.5℉ (35.83℃) at the time of admission.

Main results

Of the 57 patients enrolled in the study, 21 developed hypothermia during their stay and 36 did not. Our results show that patients who have hypothermia at the time of admission spend a longer time intubated (p < 0.01) and go through longer ICU stays (p < 0.01). These patients are also 2.18 times more likely to suffer a fatal outcome compared to patients that did not develop hypothermia while in the intensive care unit (Chi-squared = 8.6209, p < 0.01, RR = 2.18).

Conclusions

Hypothermia in patients with severe COVID-19 at the time of admission to the ICU is associated with poorer outcomes for patients. This manifests as a longer period of intubation, longer ICU stay, and increased risk of mortality.

## Introduction

The importance of hypothermia is demonstrated by the fact that it provides a key contribution to the predictive abilities of all three major scoring systems: Systemic Inflammatory Response Syndrome (SIRS), National Early Warning Score (NEWS) and the quick Sequential Organ Failure Assessment (qSOFA) score [1]. The inclusion of hypothermia in the SIRS criteria is of particular interest since it marks the onset of major organ dysfunction [2]. In fact, the presence of hypothermia in septic patients who fulfill the SIRS criteria and have evidence of an infection is associated with higher 28-day and one-year mortality [3]. This is a recurrent theme in the literature, where hypothermic patients are found to have significantly worse outcomes than normothermic and hyperthermic patients with sepsis [4,5]. Similarly, NEWS is strongly associated with intensive care admissions and hospital mortality [6]. In fact, a recent study by Jang et al. (2020) showed the NEWS score to be superior to qSOFA when predicting 28-day mortality and superior to both SIRS and qSOFA in predicting critical outcomes [7]. Despite hypothermia not being directly incorporated into the qSOFA as a metric, it has been shown that it enhances and accurately complements the predictive ability of the qSOFA in patients with sepsis at high risk of mortality [8].

The Coronavirus-19 disease (COVID-19) describes a multisystem disease with a predominantly respiratory syndrome caused by the severe acute respiratory syndrome coronavirus-2 (SARS-CoV-2) [9]. Although the majority of those infected will undergo an asymptomatic or mild disease course [9], it is estimated that one in five of those infected will require a hospital admission, with 1 in 10 needing an admission to ICUs [10]. The physiological reaction to the disease is characterized by considerable antibody production, pneumonia, cytokine storms, and lymphopenia [9]. Now over a year since the beginning of the pandemic, and with multiple vaccines having been approved for use, it has become apparent that new strains of the virus are spreading in the population, threatening the efficacy of our latest efforts to control the spread of the disease. Thus, the number of cases continues to rise, surpassing the 100 million mark globally, and highlighting the need for better management and prognostication, which will prove instrumental in guiding conversations with the relatives of those severely affected.

In light of this information, we sought to investigate the association between hypothermia and mortality in a cohort of patients suffering from severe COVID-19 and requiring an ICU admission. Gaining a greater understanding of this association is particularly important considering there is still no reliable measure of disease severity, or definitive treatment for the disease. Identifying clinical indicators of that nature will aid in decision-making and approaching goals of care conversations between patients, their families, and the medical team.

## Materials and methods

Study population

The data were gathered retrospectively from a cohort of patients who attended a community hospital servicing a large rural population in southern Indiana and eastern Illinois, and required an ICU admission between 1st of March 2020 and 30th of September 2020.

The inclusion criteria for the patients considered in the study were:

● Admission to the intensive care unit (ICU level 2 & 3).

● Any requirement of mechanical ventilation due to hypoxic respiratory failure during their hospital stay (SpO2 < 88%).

● At least one positive COVID-19 swab.

● COVID-19 listed as the official cause of death.

The exclusion criteria for the patients excluded from the study were: 

● Patients admitted to the ICU due to overflow.

● Patients admitted for observation.

● Patients directly admitted as hospice - palliative measures.

The initial pool of 67 patients identified from the hospital’s EPIC electronic medical records were narrowed down to 57 patients using the aforementioned criteria, and their outcomes formed the basis of the results of this study.

Data collection

Patient temperatures were taken orally and were recorded (every 12 hours per unit standard of care) using a standard WelchAllyn® or standard SureTemp® medical grade thermometer, with the incidence of hypothermia defined as ≥2 recorded temperatures of less than 96.5℉ [4]. Furthermore, data on age, sex, length of stay, duration of intubation, and use of an external warming method (BAIR hugger/Warm blanket) and mortality were collected for each patient upon review of individual electronic records. Ferritin levels were recorded at the time of ICU admission and used as a measure of disease severity [11]. Records were reviewed by one individual.

Statistical analysis

A one-tailed T-test was used to evaluate the relative difference in ferritin levels, length of intubation, and length of time spent in the ICU between hypothermic and non-hypothermic patients. The mortality rate of hypothermic and non-hypothermic groups was compared using a Chi-squared test, and a measure of relative risk of mortality for the hypothermic group compared to the non-hypothermic group was calculated. A Kaplan-Meier survival curve was generated to help visually present our findings.

## Results

A total of 57 patients requiring an ICU admission due to a severe COVID-19 pneumonia were included in this study. The average age of patients in this pool was 70.97 ± 10.75 with a male to female ratio of 1.54:1. In this group, 55 patients (96.49%) suffered comorbidities, with the four most common comorbidities including cardiovascular disease (77.12%), pulmonary disease (35.09%), chronic kidney disease (28.07%) and diabetes mellitus (35.09%). In both groups, over 90% of participants suffered comorbidities and there was no apparent significant difference in the rates of comorbidities between the two groups (Table 1).

**Table 1:** Baseline features and outcomes of enrolled patients. Groups were matched for sex, age and comorbidities.

	All patients, N = 57	Hypothermic group, N = 21	Non-hypothermic group, N = 36
Age, years, mean (range)	71.86 ± 10.76 (42-94)	73.71 ± 10.95 (42-94)	70.78 ± 10.65 (43-91)
Sex, male/female	34/23	12/9	22/14
Comorbidities, n (%):			
Cardiovascular	46 (77.12)	19 (90.48)	27 (75.00)
Pulmonary	20 (35.09)	8 (38.09)	12 (33.33)
Renal	16 (28.07)	9 (42.86)	5 (13.89)
Diabetes	20 (35.09)	5 (23.81)	15 (41.67)

Ferritin levels recorded at the point of admission to the ICU were used as an indication for disease severity [11]. Table 2 shows that the mean ferritin levels in the hypothermic group were almost 1.8 times those of the patients in the non-hypothermic group. The t-test value for ferritin levels between the two groups was 2.02393, P-value: 0.024281 (p < 0.05), demonstrating a statistically significant association between hypothermia and greater disease severity. 

**Table 2:** Ferritin levels for enrolled patients recorded at the point of ICU admission.

	Hypothermic group	Non-hypothermic group
Mean (μg/L)	1227.63	686.03
T-test value	t-value: 2.02393; P-value: 0.024281 (p < 0.05)	

In the group considered, 21 (36.84%) patients presented with hypothermia as defined in the methods. The remaining 36 (63.16%) did not have hypothermia recorded during their hospital stay. The average length of stay was 15.57 ± 1.87 days for the hypothermic group and 7.61 ± 0.67 days for the non-hypothermic group, and the average length of intubation was 9.00 ± 1.82 days for the hypothermic group and 0.86 ± 0.40 days for the non-hypothermic group, with both measures showing a significant difference between the groups (Table 3).

**Table 3:** Patients in the hypothermic group required external heating and stayed in the hospital for longer on average.

	All patients, N = 57	Hypothermic group, N = 21	Non-hypothermic group, N = 36	T-test values
Length of stay from ICU admission to discharge or mortality in days, mean (range)	10.54 ± 0.95 (3-32)	15.57 ± 1.87 (5-32)	7.61 ± 0.67 (3-18)	t-value: -4.76283; P-value < 0.00001 (p < 0.01)
Days of intubation, mean (range)	3.86 ± 0.88 (0-25)	9.00 ± 1.82 (0-25)	0.86 ± .40 (0-5)	t-value: -5.51573; P-value < 0.00001 (p < 0.01)
External warming device, n (%)	11 (19.30)	11 (52.38)	0 (0)	No external warming device was used in non-hypothermic patients

**Figure 1:**
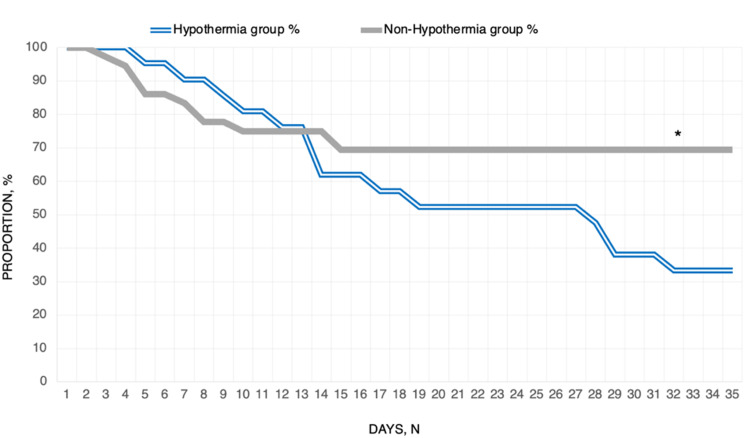
Kaplan-Meier curves for patient survival, *p < 0.01.

Of the 21 hypothermia patients, 14 (66.67%) suffered a fatal outcome due to their disease; conversely, there were 11 out of 35 (30.56%) deaths in the non-hypothermia group. The association between the presence of hypothermia and patient mortality in those hospitalized with severe COVID-19 pneumonia was shown to be statistically significant with a Chi-squared value of 8.62 and a p-value of 0.0033 (p < 0.01). Furthermore, were 2.18 times more likely to die during their admission for severe COVID-19 pneumonia if they experienced hypothermia (Table 4). The patient survival curves were plotted in Figure 1.

**Table 4:** Relationship between hypothermia and mortality.

	Total patients, n	Survived, n (%)	Deceased, n (%)	Relative risk of mortality	Chi-squared value
Hypothermic group	21	7 (33.33)	14 (66.67)	2.18	8.6209; p = 0.0033 (p < 0.01)
Non-hypothermic group	36	25 (69.44)	11 (30.56)	1	
Whole cohort	57	32 (56.14)	25 (43.86)		

## Discussion

The pathophysiology of hypothermia in SIRS and sepsis remains a mystery despite the various theories put forward to explain it. It was previously thought that hypothermia in sepsis was due to a lack of pro-inflammatory cytokines such as IL-6 and TNF-a, but studies have failed to show a depression of the inflammatory response in hypothermic sepsis [12,13]. Conversely, another proposed mechanism for the onset of hypothermia is an increased anti-inflammatory response through either increased anti-inflammatory cytokines such as IL-10, or reduced responsiveness of WBC to inflammatory signals. When this was investigated, no differences were observed in the levels of anti-inflammatory cytokines and inflammatory cell responsiveness of hypothermic and non-hypothermic patients with sepsis [14]. On the other hand, an evaluation of endothelial factors and co-morbidities of hypothermic patients showed that hypothermia is associated with an increased endothelial-derived biomarker fractalkine as well as an increase in cardiovascular comorbidities such as hypertension and chronic cardiovascular insufficiency [14]. This would indicate that the adverse outcomes associated with hypothermia may be underlined by vascular dysfunction as opposed to changes in pro-inflammatory or anti-inflammatory cytokines. The association of hypothermia with cardiovascular conditions would support this in that it may reflect the requirement for intact endothelial function in maintaining core body temperature.

The results of our study indicate that patients with severe COVID-19 warranting an ICU admission have a worse prognosis if they had hypothermia at the time of ICU admission. We chose to use ferritin as an inflammatory marker to provide some indication of disease severity in both patient groups, as it was recently shown that a higher level of ferritin was related to both disease severity and higher mortality in COVID-19 patients [11]. Our results demonstrate a significant relationship (p < 0.05) between higher ferritin levels and those patients who were hypothermic, reinforcing the notion that low body temperature has value as a predictive marker of patient outcome; more specifically, poor prognosis in patients with severe COVID-19 pneumonia warranting an ICU admission (Table 2). This poorer prognosis seems to be multifaceted. Our results show that hypothermia is associated with longer stays in the ICU, as well as longer intubation periods. (Table 3) Furthermore, we show that a larger proportion of the patients in the hypothermic group did not survive through their illness (p<0.01). In fact, patients who were hypothermic were calculated to be 2.18 times more likely to die during their admission (RR = 2.18) (Table 4). It is a good question whether cardiovascular comorbidities may explain the presence of hypothermia given their high representation in the cohort. The two study groups have similar rates of cardiovascular comorbidities (Table 1), and therefore, alone, they do not account for the difference in mortality between the hypothermic and non-hypothermic groups. 

In line with these results, we suggest that prognostication models for COVID-19 might include hypothermia, as this could help guide patient management and discussions with the family members of those affected. We also put forward compelling evidence for tighter monitoring of patients’ body temperature as this can indicate the severity of their illness. The need to include hypothermia as a prognostic marker comes at a time where new strains of the virus continue to emerge, casting doubts over the efficacy of vaccines recently developed and distributed. With some estimates pointing towards it being another six to twelve months before there is a return to normality, the development of accurate prognostic models will be instrumental in the continued effort of the medical community to fight the current pandemic. These prognostic models will allow for better identification of severe COVID-19 cases, which will help clinicians adequately manage patients and the expectations of their relatives.

Establishing a clear mechanistic relationship between hypothermia and acutely ill patients will require further investigation. The present study suffers from its small sample size, as well as from the restricted selection criteria used in identifying patients; reducing our ability to generalize these results across different settings and environments. Further studies tackling the association of hypothermia and the poor prognosis of COVID-19 patients should include a larger study cohort to strengthen the power of study and the confidence in the results presented here. A larger cohort will also increase the representation of patients with fewer or no comorbidities. This is necessary as it allows us to discern the differential contribution of hypothermia compared to other existing comorbidities, particularly cardiovascular-related dysfunction, to patient outcomes. It may help in understanding whether the pathophysiology underlying the effects of hypothermia are directly linked to the hypothermia itself or if they are a result of exacerbation of pathways triggered by the respective comorbidities. Additionally, an evaluation of whether the artificial maintenance of normothermia in hypothermic patients improves their outcomes could guide future management of cohorts similar to those presented in our study. Finally, it has been reported that among severely ill COVID-19 patients, those who are recognized early and gain timely access to medical intervention have lower risks of mortality and a better prognosis [15]. Further studies should aim to standardize this measure by collecting data on more objective criteria such as the time from onset of symptoms or first positive test, and expand the investigation to symptomatic non-ICU cohorts, as well as asymptomatic hospitalized COVID-19 patients. 

## Conclusions

Our results indicate that hypothermia at the time of ICU admission in patients with severe COVID-19 is associated with a significantly higher elevation of inflammatory markers such as ferritin. Furthermore, hypothermia is associated with longer intubation periods, longer ICU stays and increased risk of mortality. We suggest that further studies with larger patient samples confirm our findings and that hypothermia may be a useful marker of patient prognosis. 
